# Practical and Ethical Considerations for Schools Using Social Media to Promote Physical Literacy in Youth

**DOI:** 10.3390/ijerph17041225

**Published:** 2020-02-14

**Authors:** Trevor Bopp, Michael Stellefson

**Affiliations:** 1 Department of Sport Management, University of Florida, Gainesville, FL 32611, USA; 2 Department of Health Education and Promotion, East Carolina University, Greenville, NC 27858, USA; stellefsonm17@ecu.edu

**Keywords:** physical literacy, social media, ethics, health education, wearable technology

## Abstract

The rapid development of social media has led to its increased use by children and adolescents for health and well-being purposes. Accordingly, social interactions resulting from social media use can be further integrated into physical and health education pedagogy. Given the relationship between increased physical literacy and positive health outcomes, best practices and lessons learned from social media use in the healthcare industry should be adopted by health and physical educators practicing in schools. Thus, the purpose of this paper is to comment on several practical and ethical challenges and opportunities associated with using social media to improve physical literacy among youth. Specifically, two of the most prominent issues are discussed in depth: (1) integration of social media in physical education settings that educate children and adolescents about the biopsychosocial effects of physical activity, and (2) use of wearable technologies among youth to accrue experiences that enhance physical literacy competencies. In our opinion, health and physical educators who utilize the ALL-ENGAGE Playbook described in this commentary will successfully reach, engage, and impact students with popular social media that adequately promotes physical literacy, including through experiential use of wearable technologies.

## 1. Introduction

The rapid development of social media has led to its increased use by children and adolescents for health and well-being purposes [1]. Six types of social media platforms are commonly used in health education/promotion: (1) social networking (e.g., Facebook); (2) blog comments and forums; (3) microblogging (e.g., Twitter); (4) media sharing (e.g., Instagram); (5) book marketing; and (6) social news [2]. Such platforms, along with the Internet and other mobile technologies, are now being widely utilized to share health information [3]. Social media thus affords new opportunities for peer-to-peer health information exchange and self-management support, as well as digital disease detection and monitoring [4]. One in five United States (U.S.) children and adolescents are affected by obesity [5], which subsequently hinders motor skill development and physical activity levels [6,7], so it is imperative that educational systems begin to carefully consider how innovative technologies, such as social media, can help youth become more physically literate and active. Although YouTube is one of the most utilized and popular social media sites for all users, particularly for video content [8], younger populations are more likely to use Instagram and Snapchat, with 72% and 69%, respectively, of U.S. teens frequently on these social media platforms [1]. These popular social media websites are particularly useful for delivering targeted persuasive messages that can easily be acted on and shared with online social network members [2]. Furthermore, the integration of social media, technology, and consumer-driven online health information has very strong potential to revolutionize youth health and wellness education programs [9,10].

The education industry continues to transform and progress with the advancement of technology. Recently, there has been a marked increase in the number of virtual schools that capitalize on the low-cost and accessible use of web-based and mobile pedagogical tools [11]. Physical education, in particular, is currently experiencing tremendous growth in the number of classes offered through distance education platforms. Over half (*n* = 31) of U.S. states allow academic credit to be earned through participation in virtual physical education (VPE) courses [12]. Delivery of VPE courses can serve as a viable alternative for students who may travel regularly due to extracurricular activities (e.g., for the arts, music, or sports), reside in remote geographical locations, and/or have special needs or disabilities. Other ideal candidates for VPE include students who may be insecure about their physical skills or abilities, or who may want to take classes that are not offered at their own school due to a lack of certified instructors or insufficient facilities and/or fitness equipment [12,13].

As a desired outcome of physical education in the U.S. [14], physical literacy should be a primary consideration in the development of both traditional physical education and VPE courses. Physical literacy in the U.S. is defined as “the ability, confidence, and desire to be physically active for life” [15] (p. 3). Being physically literate contributes to a healthy life-course trajectory as it is both the foundation for, and enhanced by, physical activity [16]. As such, high physical literacy can help youth manage their weight and reduce health risks [17,18]. Unfortunately, many U.S. school systems and educators find it difficult to reorient and implement change in physical education to focus on enhancing physical literacy [19,20,21]. One approach used to overcome current barriers is increasing the scalability of VPE courses and online resources [22]. Programs such as VPE show promise in enhancing physical literacy among youth who are particularly vulnerable to health disparities resulting from a lack of physical activity [13].

Unfortunately, physical educators are not always comfortable with using VPE learning management systems (e.g., Blackboard, WebCT, and Moodle). Most notably, teachers express difficulty initiating and maintaining communication about physical activity with their students. They also encounter challenges monitoring students’ actual engagement in physical activity [23]. Vollum [10] put forward social media as a potential remedy for overcoming challenges faced by online instructors. Understanding that physical and health education should be “nurturing, empowering, motivating and engaging for all students” (p. 562), Vollum argues that the social interactions resulting from social media have become integrated into standard physical and health education pedagogy. Given the relationship between increased physical literacy and positive health and academic outcomes [15,16,24], it stands to reason that best practices and lessons learned from social media use in the healthcare industry be adopted by health and physical educators practicing in schools. Thus, the purpose of this paper is to consider and provide commentary on several practical and ethical challenges, as well as opportunities, for using social media to improve physical literacy among youth. Further, we introduce and suggest the use of the ALL-ENGAGE Playbook for school administrators and physical educators to consider when integrating social media and technology as pedagogical tools.

## 2. Practical and Ethical Issues

The healthcare industry has capitalized on these social media trends and other online health resources to virtually monitor and manage patients, as well as to disseminate and serve as a collaborative online resource for health information [3,4]. However, with these positive gains have come ethical concerns particular to social media applications. Previous work completed to assess online physical literacy resources, such as YouTube, revealed not only a desire for quality physical literacy information and content, but also an online community with high standards that places value on such information [25]. Physical education instructors in school systems should be cognizant of these expected quality standards. As standards evolve over time, they will undoubtedly affect the potential of social media to enhance virtual student-to-student, student-to-teacher, and student-to-industry networking opportunities, as well as the availability, accrual, and dissemination of physical literacy information and resources for students.

McKee [3] has described ethical issues of privacy, anonymity, data collection, and patient surveillance on social media. Issues such as these sparked the need for new progressive ethical standards for using social media in health care [9]. Denecke [4] discussed ethical concerns associated with the increased use of web-based technology and social media in healthcare settings. With these concerns in mind, we now consider the following two critical issues most closely associated with utilizing social media to improve youth physical literacy in schools: (1) the integration of social media in elementary, middle, and high school settings that educate children and adolescents about physical literacy, and (2) the use of wearable technologies among these youth during physical education to learn more about physical literacy competencies.

### 2.1. Integration of Social Media in Physical Education Settings that Educate Children and Adolescents about Physical Literacy

The inherent practical and ethical challenges associated with youth using these popular social media websites and applications (apps) include problems with obtaining consent from their parents or guardians, as well as the privacy of youth and their level of comfort sharing personal information online [4] about their own physical literacy (e.g., body, motor skills, movement, physical activity and fitness, and value and accountability). Physical literacy can be a very personal and individualized journey [26]. One primary benefit of VPE and social media usage is the provision of a safe and potentially private space for students who may be insecure about their physical skills and abilities [13]. Thus, it is critical that educational protocols and policies be explicit regarding storage and usage of, and access to, student data. Students and parents/guardians should also be fully informed about, and understand clearly, that to which they are consenting [27].

However, administrators should be accountable for and concerned not only about the data and student-created content that is collected and stored. Use of misleading educational physical and health information on social media can be very dangerous for youth and the spread of unverified health claims can also be very harmful. Nevertheless, sharing personal experiences to establish meaningful person-to-person connections is central to the social media experience and has the potential to empower users to become more activated in terms of health information-seeking and health-related decision-making [28,29]. Physical education teachers who elect to incorporate social media into the classroom environment should be conscientious about how social media is used to mitigate barriers to youth physical literacy that may exacerbate existing health disparities.

Regarding this practical and ethical challenge, it is imperative that social media platforms be reconfigured and adapted as an educational tool for communication between students and teachers. A common misunderstanding about social media is that it serves as a podium for delivering a message. Instead, social media enables teachers to talk with their students rather than simply talking at them. Talking with students on social media helps to build a sense of trust and adds a personal touch [2]. However, student–teacher online relationships can be ethically questionable and should be maintained in a safe, trusting, and professional manner [4].

Social media should provide engaging, supportive, applicable, and practical physical literacy information and resources. For example, using images and videos of role model child/adolescent actors taking part in healthy physical activity is more likely to capture students’ attention and enhance message comprehension. Posts with visual images also typically receive more clicks, shares, comments, and likes on social media [30,31]. However, the applicability, acceptability, and reach of social media posts is often limited due to the large number of posts constantly being made on popular social media channels. Conversely, if educators do not attentively monitor students’ social media conversations, these conversations can quickly become unpredictable and result in confusion and frustration for the students [32]. Likewise, if educators do not consistently add and update physical and health education content, the social media sites they manage are likely to lack robust and informative conversations [33].

Currently, there are few models available to health and physical education specialists that describe guidelines for planning, implementing, and evaluating social media pages for health promotion and disease management. Therefore, a new social media awareness, outreach, and engagement “playbook” was developed to foster meaningful interaction with priority audiences [2]. The ALL-ENGAGE Playbook [34] is a social media framework, inspired by evidence-based social media guidelines, including those provided by the Centers for Disease Control and Prevention (CDC) [35,36,37], that should be considered by physical education teachers and programs to establish and facilitate worthwhile participation and discussion with students. This would involve: assessing social media channels to best reach student(s); listening to social media conversations to identify trending physical activity and literacy topics; leveraging existing gaps and opportunities for using social media in physical education; editing course posting calendars to manage social media administration; networking with peers who have similar physical activity/education interests; generating new social media content that is search engine optimized (SEO); adapting social media policies to facilitate interactivity and diverse opinions; guiding students to current, accurate, and evidence-based physical activity and health/wellness information; and evaluating social media pages through systematic analysis of performance indicators. Table 1 outlines plays, action steps, and goals associated with each element of the ALL-ENGAGE Playbook.

**Figure 1:**
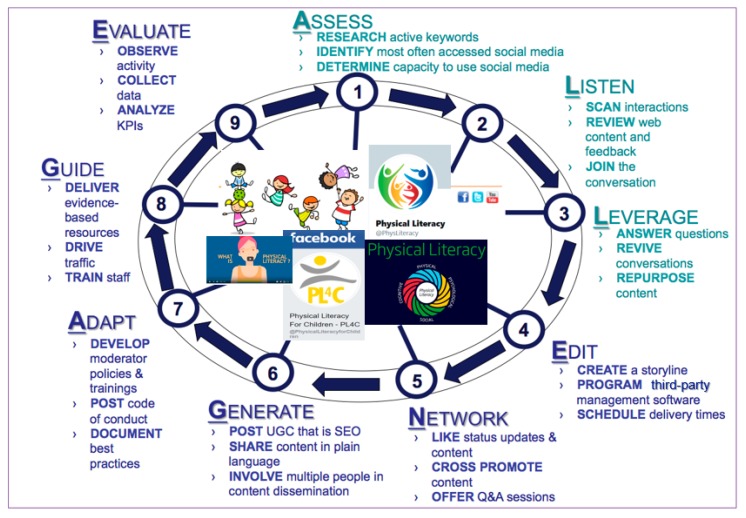
ALL-ENGAGE Playbook for advancing youth physical literacy on social media.

Figure 1 demonstrates the cyclical nature of the ALL-ENGAGE Playbook strategy, with a focus on the use of social media to build physical literacy competencies. Systematically planning for social media use in physical education is important given that prior work acknowledges increased risks for negative outcomes such as breaches of student confidentiality and unprofessional student-to-student or student-to-teacher interactions [4]. For example, educators who use social media to improve physical literacy should seek to remove all personally identifying student information from all posted content [2]. It is also important to monitor privacy settings on social media to limit the exposure of content outside of students within the class. Personal and professional social media profiles should be separate, with declared conflicts of interest made clear [2]. Additionally, anyone using social media tools should be required to have specific training. When preparing training for teachers and students, topics should include hashtag use, uploading content, updating profile pictures, and sharing media [40]. Educators should also be trained to remove destructive posts and students who violate stated social media policies [41]. They should additionally encourage youth to avoid potentially harmful material on social media (e.g., misguided instructions on how to exercise); consider the consequences of taking social media actions before posting, commenting, or sharing content with others; report derogatory comments pertaining to a person’s gender, race, or religion; and stay cautious of commercially motivated objectives, such as selling unregulated fitness products [42].

Daum and Buschner [23] found that teachers had a difficult time offering immediate feedback and constructive visual cues on students’ movement and physical activity assignments and this hindered both the applicable teaching and in-depth learning of motor skills. Social media apps might not currently be capable of addressing such issues. Thus, social media pedagogy should adapt teaching capabilities to meet the academic needs and learning styles of today’s youth, particularly as it pertains to social interactions in modern culture [10]. Required training would need to be developed in accordance with the ALL-ENGAGE social media playbook (Figure 1). Training teachers to become active communicators and disseminators on social media may help address parental perceptions that social media contains mostly inaccurate and biased information. Schools should adopt policies and administer training programs continuously on a semester-by-semester basis to focus on appropriate and ethically responsible use of social media in physical education and VPE.

### 2.2. The Use of Wearable Technologies among Youth

Young peoples’ understanding, use, and need for digital physical activity and health technologies varies and is greatly influenced by schools, physical education, sports, family, and peers [43]. Thus, the use of such digital physical and health technologies in the school system can be very beneficial to students’ physical literacy development, in both traditional physical education settings and VPE classes, yet presents similar, previously mentioned, ethical concerns with issues of data security, accountability, and privacy. To this end, the Guide phase of the ALL-ENGAGE playbook encourages the utilization of evidence-based digital activity and health resources and technologies. Further, the Evaluate phase describes means by which such tools can be evaluated to determine their impact on youth physical literacy and related outcomes. Wearable health technologies and trackers are typically designed for the general user to monitor particular health metrics (e.g., heart rate, steps, distance traveled) and connect to a database, via phone or internet, to allow users to manage, learn, and adjust their physical activity accordingly based on their shared data [44]. Such information would be valuable to students and teachers as it would provide them with more opportunities for varied and in-depth charting of their physical literacy journey [16,45]. Other software and technologies, such as augmented and virtual reality, can be utilized by mobile devices to aid in student development of physical literacy. It has been found that augmented reality applications, when integrated into cell phones, had a positive impact on physical education students’ learning and advancing their spatial awareness and distance estimation [46]. Taken together, integrating and cross-referencing the data collected from wearable technologies and social media sites allows educators to better understand the impact of social interactions, spatial location and environment, psychological states and affect, and other behaviors on student physical activity levels and engagement [47].

Concerns with privacy, security, and accountability have previously been discussed, but it is important to note other potential issues resulting from the use of social media and mobile technologies. For instance, wearable technologies have been found to be helpful in detecting early indications of youth’s mental health, but involved real-time and immediate monitoring of social media, sleep, and activity, which required end-users’ trust and active engagement with administrators [48]. Kerner and Goodyear [49] found evidence that wearing such devices negatively impacted physical competence and perceived autonomy due to daily activity targets (e.g., 10,000 steps per day), as well as contributed to amotivation towards physical activity and healthy lifestyles. Lastly, we must remark on the significance of access and equity in the wearing of digital activity trackers among students from marginalized communities. Requiring and/or capitalizing on the use of wearable technologies can have unintended consequences that may exasperate discrepancies in schools’ financial and community resources for youth, as well as (a lack of) conducive environments and time for youth to be physically active [50,51]. Parameters should thus be put into place that will afford all students from the same schools or districts the opportunity to participate in VPE and/or the use of wearable and mobile educational technologies, along with a transparent protocol that will monitor students’ mental and affective experiences with these devices.

## 3. Conclusions

While social media may become the way of the future for conveying and disseminating physical education to youth, its use is largely unregulated, with limited oversight provided by trained professionals. As social media becomes more integrated into school-based physical education, teachers will likely play an important role in monitoring information that is being shared. In this capacity, school administrators should engage with the public to address physical activity and health-related misconceptions or misinformation on social media in a manner that is non-confrontational and enlightening. The purpose of this paper was to consider and provide commentary on several practical and ethical challenges and to discuss opportunities for using social media to improve physical literacy among youth. To this end, we summarized two of the major practical and ethical issues surrounding the use of social media in physical education settings where physical literacy is promoted. The use of social media and wearable physical activity and health-related technologies in such settings raises some concerns with respect to privacy, security, trust, and accountability, but should be considered due to unprecedented opportunities for educating youth about the principles that guide physically literate and healthy lifestyles. While there is no one-size-fits-all approach to the use of such web-based applications and mobile devices, we introduce and suggest the use of the ALL-ENGAGE Playbook for consideration by school administrators and physical educators when integrating social media and technology as pedagogical tools. While the ALL-ENGAGE Playbook has yet to be tested in social media research among children and adolescents, we feel that there is great potential in implementing this innovative framework to promote physical literacy in youth populations. Moreover, physical educators who choose to utilize the ALL-ENGAGE Playbook in practice may be more likely to successfully reach, engage, and impact youth with social-media-based programming that enables students to learn about and enhance their physical literacy, especially through use of wearable technologies.

## Figures and Tables

**Table 1:** Goal-Directed Plays and Action Steps in the ALL-ENGAGE Social Media Playbook.

Play	Action Steps	Goal(s)
Assess social media channels	Research active keywords Identify social media websites regularly accessed (daily or almost daily) by students and youth Determine capacity to use social media	Understand students’ current and potential usage of popular social media for physical literacy, physical activity, and health/wellness information
Listen to social media conversations	Manage and monitor social media interactions between and among students (e.g., discussions, posted comments, pictures and videos) Review posted content and feedback left on relevant websites or blogs [38] Join the conversation	Identify trending physical literacy, physical activity, and health/wellness information, discussion topics, concerns, and questions Describe student interests, knowledge, physical and health literacy, and cultural perspectives on physical activity and education
Leverage existing gaps and opportunities	Answer student questions on social media Address discussions that never concluded Repurpose evidence-based physical education content to start a new conversation on social media	Make social media a valuable space for student-to-teacher interaction and communication Address physical activity and literacy management questions and concerns
Edit course posting calendars	Create a story board that personalizes and humanizes posted physical education materials and content Program third-party social media management software Schedule times for when a message is to be sent out on any day or time of the week	Post to a social media site (e.g., Instagram, YouTube, Facebook) at least three times per week, with 3-5 h per week spent engaging with users Post to Twitter or Snapchat at least once daily, with 3–5 h a week spent managing feeds
Network with students and other social media users	“Like” status updates and comments Cross promote content from reputable sources, agencies, and influencers [35] Offer Q&A social media sessions with physical activity experts	Connect students with industry professionals and other educational social media users who share similar physical activity and health/wellness interests
Generate new social media content	Post user-generated content (UGC) that is search engine optimized (SEO) Share content in language understood and used by students Involve student group work whenever possible	Increase physical activity and literacy information accessibility that reinforces important content for students and other potential stakeholder audiences Increase the frequency of interactions and improve user engagement
Adapt social media policies	Develop social media moderator policies and trainings [39] Post a social media code of conduct and grading rubric for students Document best practices for posting, moderating, and communicating on social media	Facilitate student-to-student and student-to-teacher interactivity and engagement Maintain healthy and informative participation, with a diversity of opinions related to cultural, spiritual, and political beliefs and opinions
Guide student users	Deliver physical activity and health/wellness resources Drive students to useful, interesting, and action-oriented posts Train teachers and administrators to remove destructive posts that may personally attack others	Create brand (i.e., course) recognition Engage students and discuss current topics and issues that they find relevant
Evaluate social media pages	Observe social media page activity Collect and process evaluation data Interpret key performance indicators on social media [38]	Monitor progress toward reaching short-term, intermediate, and long-term physical literacy Learn from mistakes and make program modifications accordingly
